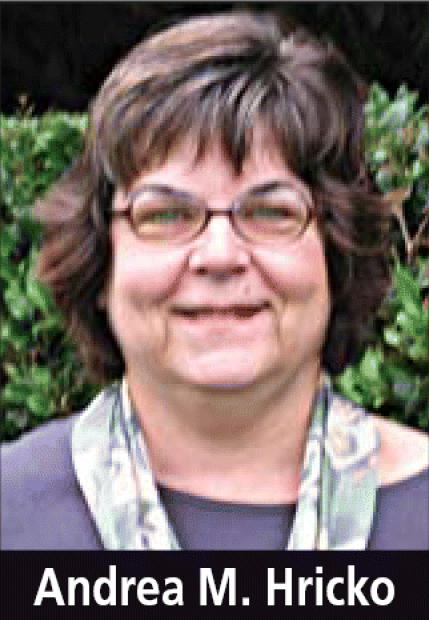# Guest Editorial: Ships, Trucks, and Trains: Effects of Goods Movement on Environmental Health

**DOI:** 10.1289/ehp.114-a204

**Published:** 2006-04

**Authors:** Andrea M. Hricko

**Affiliations:** Keck School of Medicine, University of Southern California, Los Angeles, California, E-mail: ahricko@usc.edu

Globalization is changing the world in ways that we may not yet fully comprehend. For the United States, the enactment of new free trade agreements, the downsizing of our manufacturing base, and consumer demand for inexpensive products are all affecting both jobs and the environment, especially in those regions with ports and transportation corridors designed to distribute imported goods. The changing dynamics of trade prompted a journalist to remark last month that the United States “is becoming nothing more than a distribution economy, importing, moving and selling consumer goods” (Romans 2006).

As this shift in the world and U.S. economies occurs, little attention has been placed on its environmental impacts, especially the health impacts of air pollution from international trade and “goods movement.” As the Focus article describes in this issue of *EHP* (Sharma 2006), the volume of imports from Asian countries into the United States has skyrocketed. The distribution of these goods from their entry ports to the rest of the United States involves diesel-powered vehicles and equipment every step of the way, creating significant exposures and health impacts in communities along the distribution routes that are just beginning to be assessed.

For example, a $9.97 doll is made in Asia by low-wage workers under conditions that may subject them to a myriad of unregulated hazards. This doll is packed with 10,000 others into a container and loaded onto a marine vessel holding 4,000 other containers carrying dolls, shoes, and electronics. Fueled by low-quality bunker fuel, the ship leaves one of the world’s largest ports in Asia, chugs across the Pacific, discharging nitrogen oxides, sulfur oxides, particulates, and other pollutants into the earth’s environment. Arriving at the Southern California ports of Los Angeles or Long Beach (where 40% of all U.S. imports arrive), the container is unloaded by longshore workers, who breathe exhaust from the idling ship as well as emissions from a row of idling trucks with drivers waiting for their loads. The next leg of the trip is via truck to a rail yard, situated less than one-quarter of a mile from schools and homes, where the container is placed on a freight train, pulled by a diesel locomotive. Alternatively, the doll may be placed on a big-rig truck and sent for repackaging to a mega-warehouse 50 miles from the ports, an area that was formerly all dairy lands that has now given way to million-square-foot warehouses for consumer goods (drawing thousands of diesel trucks a day into formerly rural communities). Finally, the doll is trucked to her destination, a big-box retailer in suburban Chicago. By this time, she has traveled more than 8,000 miles—on diesel-burning conveyances the whole way.

This itinerary is not unusual for shipping. Today, nearly half of all imported goods sold in Chicago take a route like this from factories in Asia through Southern California ports before heading east. But the low price a mother in Chicago pays for her daughter’s toy reflects none of the human and environmental tolls (referred to as the “externalities of transportation”) that the doll’s manufacture and shipment have taken during its travels. These include tolls on

- The world’s climate, in terms of emissions that may impact global warming
- The workers who made the doll in Asia, where occupational health and safety rules are more lax than in the United States (Wang and Christian 2003) and where wages are a fraction of U.S. wages
- Dock workers, truck drivers, and railroad workers, who may have elevated rates of lung cancer (see, for example, Garshick et al. 2004)—the basis for California declaring diesel particulate a toxic air contaminant in 1998, requiring regulations to reduce risk of exposure
- Residents in communities adjacent to truck-congested freeways, where elevated levels of carbon monoxide, diesel constituents, and ultrafine particles have been documented (Zhu et al. 2002)
- Residents living near ports, in whom there are elevated rates of oropharyngeal cancer and certain lung cancers, according to an analysis of cancer by census tracts in Los Angeles County (Mack 2004)
- Residents who breathe ambient air pollution full of traffic-related pollutants, in whom there are higher rates of cardiovascular disease and death (Jerrett et al. 2005) and reduced lung function (Gauderman et al. 2004)
- Residents who live near rail yards, ports, and other goods movement facilities, who endure high noise levels, traffic congestion, visual blight, and other community impacts
- Infrastructure (marine terminals, highways, bridges, rail lines, and rail facilities), which must be repaired or expanded, often at taxpayers’ expense, to keep pace with the surging imports.

The burden of disease from transporting imported goods longer and longer distances is growing, at both U.S. and overseas ports. According to the California Air Resources Board (CARB), the agency that regulates air pollution in California,

Air pollution from international trade and goods movement is a major public health concern at the statewide, regional and community level. Adverse health impacts from the pollutants associated with goods movement include but are not limited to premature death, cancer risk, respiratory illnesses, and increased risk of heart disease…. Adverse birth outcomes, effects on the immune system, multiple respiratory effects, and neurotoxicity are additional potential health effects. (CARB 2005a)

Also, evidence is growing that low-income, minority communities are disproportionately impacted:

Health risk at the community level is of special concern because exposure is highest near ports, rail yards, and along high volume truck traffic. The Californians who live near ports, rail yards, and along high traffic corridors, are subsidizing the goods movement sector with their health. (CARB 2005b)

Surely, one asks, these problems must be solved by strict emission controls on ships, trains, and trucks and the ports and rail facilities they traverse. Surprisingly, no, say air pollution regulators. According to the South Coast Air Quality Management District (2005), the agency that regulates air pollution in Southern California, *a*) more than 90% of oceangoing vessels calling on U.S. ports are foreign-flagged, with emissions covered by weak International Maritime Organization standards and no U.S. Environmental Protection Agency (EPA) controls; *b*) federal emission rules for locomotives are more lenient than for other emission sources, and new U.S. EPA rules have not yet been issued; *c*) emission rules for trucks are in effect, but some old truck engines will be on the road for decades; and *d*) although some ports are working on new emission control programs, ports continue to be sources of large and growing quantities of emissions.

This situation calls out for stricter local, state, national, and international rules to protect workers and residents from the health effects of air pollution. It also calls for more epidemiologic and exposure assessment studies, as well as sophisticated cost–benefit analyses, of the impact that promotion of international trade and goods movement is having on residents’ and workers’ health—and whether being a “distribution economy” is the best strategy for the U.S. economic future [see, for example, economic questions raised by Haveman and Hummels (2004)].

Such an analysis would need to include *a*) externalized health costs of air pollution, including all health end points; *b*) the cost of loss of manufacturing jobs and benefits of goods movement jobs; and *c*) other community impacts (noise, aesthetics, traffic congestion, accidents, and costs of expanding infrastructure to handle rising imports).

The issue of international trade, ports, and goods movement lies at the intersection of globalization, economics, transportation, land use planning, sustainability, and health. An environmental health research funding partnership could help bring these diverse interests together as a means of documenting health impacts and searching for public health solutions. Such an innovative effort could be led by the National Institute of Environmental Health Sciences (NIEHS) and involve, at least, the U.S. EPA, the Department of Transportation (including its Federal Highway, Federal Railway, and Maritime Administrations), the Department of Commerce, the Department of Labor, the Office of the U.S. Trade Representative, and the Transportation Research Board of the National Academies.

Finally, as transportation and elected officials around the country call for expanding the nation’s infrastructure (ports, marine terminals, highways, rail lines, and facilities) to promote growth in international trade, there is an urgent need—and a challenge—for “health” to become a more central part of the policy discussion.

## Figures and Tables

**Figure:**